# P-1632. Effectiveness of remdesivir in patients with underlying hepatic or renal comorbidity hospitalized for SARS-CoV-2 infection

**DOI:** 10.1093/ofid/ofaf695.1808

**Published:** 2026-01-11

**Authors:** Patrick Godwin, John Jay Hawkshead, Valentina Shvachko, Thomas F Oppelt, Chen-Yu Wang, Essy Mozaffari, Amos Lichtman, Mark Berry

**Affiliations:** Department of Medicine, University of Illinois College of Medicine, Chicago, Illinois; Gilead Sciences, Inc., Foster City, California; Gilead Sciences, Inc., Foster City, California; Gilead Sciences, Inc, Foster City, California; Gilead Sciences, Inc., Foster City, California; Gilead Sciences, Inc., Foster City, California; Gilead Sciences, Inc., Foster City, California; Gilead Sciences, Inc., Foster City, California

## Abstract

**Background:**

Individuals infected with SARS-CoV-2 with underlying hepatic or renal comorbidities are at higher mortality risk than the overall population. Antiviral treatment with remdesivir (RDV) in individuals hospitalized for SARS-CoV-2 has been shown to reduce in-hospital mortality across many high-risk groups, regardless of COVID-19 severity and variant era. Data on the effect of early RDV treatment in these high-risk subpopulations are needed to inform clinical decision-making.

**Figure:**
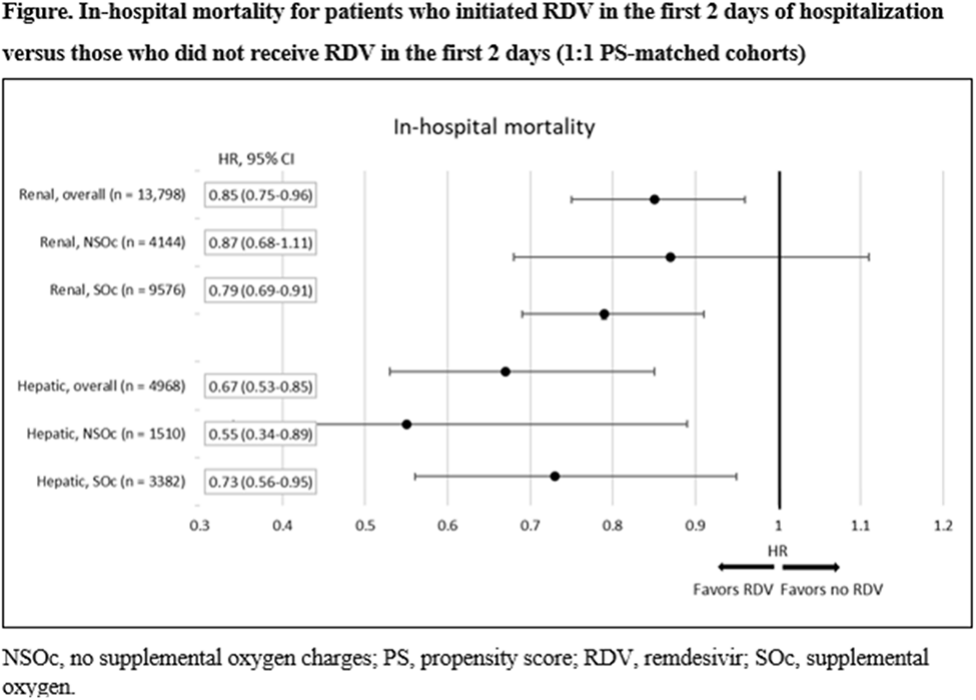

**Figure:**
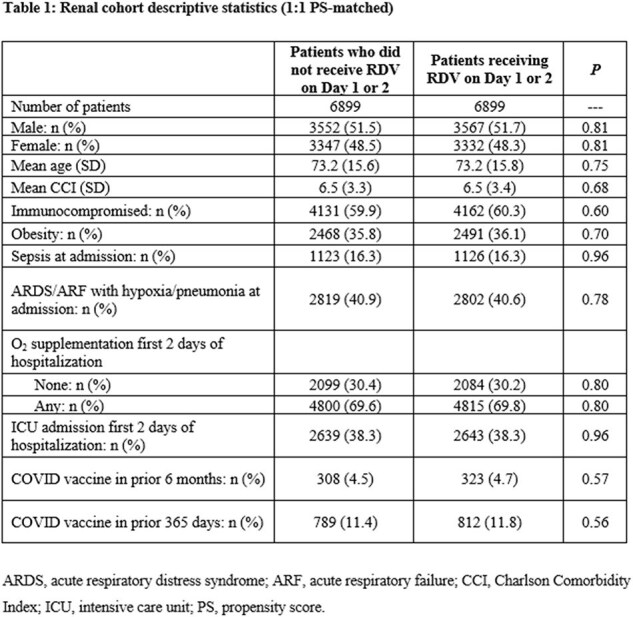

**Methods:**

This retrospective study of U.S. medical claims and hospital chargemaster data examined the effectiveness of initiating RDV upon hospital admission, in the first 2 days, on all-cause mortality or discharge to hospice among adults hospitalized for SARS-CoV-2 infection with pre-existing hepatic or renal comorbidity, vs no RDV treatment in the first 2 days. Patients were followed for up to 30 days, until discharge, or death, whichever occurred first. Coarsened exact matching and 1:1 propensity score (PS) matching were based on demographic, clinical, and hospital characteristics. Mortality risk was compared via Cox proportional hazard ratio (HR) and 95% confidence interval (CI). Analysis was stratified by supplemental oxygen (O_2_) support received during the baseline, in the first 2 days.

**Figure:**
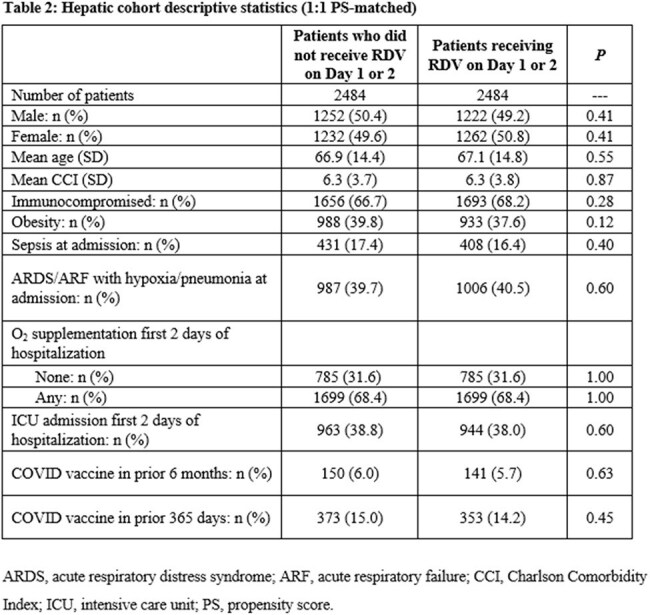

**Results:**

The 1:1 PS-matched renal cohort included 13,798 patients (mortality, 6.05%; Table 1) ; the 1:1 PS-matched hepatic cohort included 4,968 patients (mortality, 4.59%; Table 2). Remdesivir use was associated with lower risk of in-hospital mortality in each cohort overall—renal 0.85 (95% CI 0.75, 0.96), hepatic 0.67 (0.53, 0.85)—as well as among more severely ill patients in each cohort requiring supplemental O_2_ upon admission: renal 0.79 (0.69, 0.91), hepatic 0.73 (0.56, 0.95). Hepatic patients who did not require supplemental O_2_ also had significantly lower mortality risk: 0.55 (0.34, 0.89). Mortality risk reduction in renal patients not requiring supplemental O_2_ was not statistically significant: 0.87 (0.68, 1.11).

**Conclusion:**

Individuals hospitalized with SARS-CoV-2 infection with underlying hepatic or renal comorbidity treated with RDV realized in-hospital survival benefit over those not similarly treated, both overall and in severely ill patients receiving supplemental O_2_.

**Disclosures:**

Patrick Godwin, MD, Gilead Sciences, Inc.: Advisor/Consultant|Gilead Sciences, Inc.: advisory board, speakers bureau John Jay Hawkshead, DrPH, Gilead Sciences, Inc.: Employee|Gilead Sciences, Inc.: Stocks/Bonds (Public Company) Valentina Shvachko, MS, Gilead Sciences, Inc.: Employee|Gilead Sciences, Inc.: Stocks/Bonds (Public Company) Thomas F. Oppelt, PharmD, BCPS, Gilead Sciences, Inc.: Employee|Gilead Sciences, Inc.: Stocks/Bonds (Public Company) Chen-Yu Wang, PhD, Gilead Sciences, Inc.: Employee|Gilead Sciences, Inc.: Stocks/Bonds (Public Company) Essy Mozaffari, PharmD, MPH, MBA, Gilead Sciences, Inc.: Former Employee|Gilead Sciences, Inc.: Stocks/Bonds (Public Company) Amos Lichtman, MPH, MD, Gilead Sciences, Inc.: Employee|Gilead Sciences, Inc.: Stocks/Bonds (Public Company) Mark Berry, PhD, Gilead Sciences, Inc.: Employee|Gilead Sciences, Inc.: Stocks/Bonds (Public Company)

